# Microbial functional genes are driven by gradients in sediment stoichiometry, oxygen, and salinity across the Baltic benthic ecosystem

**DOI:** 10.1186/s40168-022-01321-z

**Published:** 2022-08-15

**Authors:** Elias Broman, Dandan Izabel-Shen, Alejandro Rodríguez-Gijón, Stefano Bonaglia, Sarahi L. Garcia, Francisco J. A. Nascimento

**Affiliations:** 1 Department of Ecology, Environment and Plant Sciences, Stockholm University, 106 91 Stockholm, Sweden; 2 Baltic Sea Centre, Stockholm University, Stockholm, Sweden; 3 Science for Life Laboratory, Stockholm, Sweden; 4 Department of Marine Sciences, University of Gothenburg, Gothenburg, Sweden

**Keywords:** Metagenomics, Benthic, Metabolism, Microbial, DNA, Bacteria

## Abstract

**Background:**

Microorganisms in the seafloor use a wide range of metabolic processes, which are coupled to the presence of functional genes within their genomes. Aquatic environments are heterogenous and often characterized by natural physiochemical gradients that structure these microbial communities potentially changing the diversity of functional genes and its associated metabolic processes. In this study, we investigated spatial variability and how environmental variables structure the diversity and composition of benthic functional genes and metabolic pathways across various fundamental environmental gradients. We analyzed metagenomic data from sediment samples, measured related abiotic data (e.g., salinity, oxygen and carbon content), covering 59 stations spanning 1,145 km across the Baltic Sea.

**Results:**

The composition of genes and microbial communities were mainly structured by salinity plus oxygen, and the carbon to nitrogen (C:N) ratio for specific metabolic pathways related to nutrient transport and carbon metabolism. Multivariate analyses indicated that the compositional change in functional genes was more prominent across environmental gradients compared to changes in microbial taxonomy even at genus level, and indicate functional diversity adaptation to local environments. Oxygen deficient areas (i.e., dead zones) were more different in gene composition when compared to oxic sediments.

**Conclusions:**

This study highlights how benthic functional genes are structured over spatial distances and by environmental gradients and resource availability, and suggests that changes in, e.g., oxygenation, salinity, and carbon plus nitrogen content will influence functional metabolic pathways in benthic habitats.

Video Abstract

**Supplementary Information:**

The online version contains supplementary material available at 10.1186/s40168-022-01321-z.

## Background

Aquatic microorganisms in the seafloor use a range of metabolic pathways to produce, degrade, or transform nutrients and biogeochemical elements [1]. Metabolic processes are dependent on the presence of functional genes within genomes coding for required enzymes and transporters. The community assembly of aquatic microbes, and consequently the functional genes they carry, can show strong spatial heterogeneity that stem from both deterministic and stochastic processes [2, 3]. Deterministic processes often shape microbial communities by driving adaptations to local environmental conditions [4]. As such, environmental gradients have been found to be important for the community structure of aquatic microorganisms [2–6] and can be expected to also impact metabolic capabilities in marine sediments. Gradients in, e.g., salinity, organic matter content, temperature, and oxygen conditions, occur naturally in aquatic ecosystems. In addition, anthropogenic impacts to the environment alters the strength of such environmental gradients [5–9], with consequent impacts on the structure of benthic microbial communities [10–12]. For example, changes in freshwater runoff are expected to increase the extent of low-saline areas in enclosed water bodies such as the Baltic Sea [8]. This is important because salinity can structure the community composition of micro- and macroorganisms [13–19]. Differences in environmental conditions over spatial distances might therefore influence the diversity and composition of functional genes and metabolic processes.

Previous studies investigating functional gene diversity in benthic environments collected sediments from few sampling sites (< 10) [20–24] likely due to the labour intensive and complex logistics sampling sediments. Conversely, great effort has been done to investigate functional gene diversity in the water column on a global scale [25]. The benthic environment is understudied compared to pelagic water, even though sediment might harbor 3–4 orders of magnitude more microorganisms per milliliter [26], and their metabolisms are known to respond to anthropogenic pressures including climate change [10, 11, 27, 28]. Interestingly, settling particles from the water column are known to harbor microbial communities and might contribute to changes in gene diversity and abundance in the sediment surface [29, 30]. It has been shown in experimental studies that carbon and nitrogen resources (carbohydrates, proteins, or nucleic acids) as well as the compound structure (monomer and polymer) influences the transcriptional activity of functional genes of marine bacteria [31]. Furthermore, the type of dissolved organic carbon can select for specific microbial populations with some bacteria being potential specialists for specific compounds [31, 32]. Considering the many different types of organic matter and the associated microorganisms utilizing these substrates that are found globally in sediments, changes in carbon and nitrogen content might be reflected in the diversity and composition of functional genes at large spatial scales. Benthic environments are therefore complex ecosystems, and it remains unclear which specific environmental drivers structure functional gene diversity and metabolic pathways in marine sediments. The Baltic Sea, with its pronounced environmental gradients, in salinity, nutrient loads and oxygen conditions is an ideal model system for studying microbial communities over spatial distances and their responses to environmental change [33].

Here, we present findings from metagenomic data of sediment samples with related abiotic data collected between May and June 2019 from 59 stations covering 1145 km (Baltic Sea) including gradients in water depth, salinity, temperature, O_2_, and total carbon (TC) and total nitrogen (TN) availability and isotopic signatures. We aimed to investigate the environmental variables structuring the diversity and composition of functional genes and metabolic pathways. We hypothesized that changes in environmental conditions such as lower oxygen availability, decreased salinity, and lower TC & TN content limit benthic functional capabilities by decreasing the diversity and changing the composition of functional genes.

## Methods

### Field sampling and study site

The top 2 cm of sediment was collected at soft bottom clay-muddy habitats from 59 stations from north to south in the Baltic Sea during May 10–June 4 2019 (see Data S1 for a full list of sampling dates and WGS84 coordinates). The Baltic is a brackish water system with a shallow mean water depth of 57 m [15]. In the north, salinity is close to freshwater conditions (< 2) that gradually increase to ca 17 in the south [15]. The Baltic Sea has been heavily eutrophicated during the last ~ 50 years and today contain large bottom areas being oxygen-deficient [9]. The samples were collected by the Swedish National and Regional Benthic Monitoring Program [34], and the stations were numbered from 1 to 59 based on a decrease in latitude coordinates. The stations were grouped into regions based on geographic location and known Baltic Sea basin bathymetry, with stations below the Bothnian Sea sharing the same locations and regions as presented in Broman E, Raymond C, Sommer C, Gunnarsson JS, Creer S and Nascimento FJA [16]. One sediment core was collected per station using a Kajak gravity corer (surface area: 50 cm^2^, one core per station) and the top 0–2 cm layer was sliced into a 215-ml polypropylene container (207.0215PP, Noax Lab, Sweden). The sediment was homogenized and stored at – 20 °C on the boat, kept on iced for ~ 2 h during transportation to the university, and finally stored again at – 20 °C until DNA extraction. Bottom water (~ 20 cm above the sediment surface) was collected at each station with a Niskin bottle. This was followed by on deck measurements of bottom water temperature, salinity, and dissolved O_2_ using a portable multimeter (HQ40D, Hach).

### Sediment chemical and isotope analyses

From each sample 1.5 mL sediment was dried at 60 °C for measurements of total carbon (TC), total nitrogen (TN), and stable ^13^C and ^15^N isotope compositions. The analyses were performed with an elemental analyzer (Europa EA-GSL, Sercon Ltd., Cheshire, UK) coupled to an isotope ratio mass spectrometer (20-22 IRMS, Sercon Ltd.). Concentrations of solid phase TC and TN are reported as % by mass, and their ratio denoted as C/N. Isotopic compositions are reported using the conventional delta notation, which reports the isotopic composition of a sample as the ‰ deviation of a sample relative to atmospheric N_2_ (δ^15^N ‰) and relative to PDB (δ^13^C ‰) according to our previous protocols [35].

### DNA extraction and sequencing

The sediment samples were thawed, homogenized, and a subsample of 0.25 g was used for DNA extraction using the DNeasy PowerSoil kit (Qiagen) according to the manufacturer’s protocol. The quantity and quality of eluted DNA were measured using NanoDrop One spectrophotometer and Qubit 2 (both by ThermoFisher Scientific) to ensure that samples meet the minimum requirements for sequencing. The samples were then sequenced at the SciLife laboratories facility on two NovaSeq 6000 S4 lanes using a 2 × 150 bp setup. Sequencing yielded on average 53.0 million reads per sample (min 31.6, max 99.3). See Data S2 for a full list of station labels, fastq file names, and the number of sequences retrieved.

### Bioinformatic analyses—quality trimming

Illumina adapters were removed from the raw read data using SeqPrep 1.2 and targeting the adapter sequences [36]. Removal of any leftover PhiX control sequences were conducted by mapping the data onto the PhiX genome (NCBI Reference Sequence: NC_001422.1) using bowtie2 2.3.5.1 [37]. The reads were then quality trimmed using Trimmomatic 0.36 with settings: LEADING:20 TRAILING:20 MINLEN:80 [38]. The results from the quality trimming were verified with FastQC 0.11.8 [39] and MultiQC 1.9 [40]. The final quality trimmed data (trimmomatic “paired without unpaired” output, PwU) consisted on average of 47.0 million reads (min 28.5, max 87.9), with an average length of 137 bp with a Phred33 quality score of 36 (see Data S2 for full details per sample).

### Bioinformatic analyses—read annotation

For functional gene analysis a protocol similar to the SAMSA2 pipeline was used [41]. In more detail, the R1 and R2 reads were merged using PEAR 0.9.10 using default settings [42]. After merging the data consisted of an average of 39.1 million reads (min 23.1, max 69.7) with an average length of 164 bp. The merged reads were classified against NCBI NR (database downloaded May 30, 2020) using DIAMOND 2.0.4.142 with default settings, i.e., *e* value threshold 0.001, up to 25 hits per read query, and use of the tantan repeat masking algorithm to remove spurious hits [43, 44]. The output .daa files were meganized (i.e., accession numbers linked to NCBI taxonomy and KEGG KO identifiers) using the tool daa-meganizer supplied with the software MEGAN Ultimate Edition version 6.20.17 [45] using the software supplied megan-map-Jan2021-ue.db database. Here, we used the default settings with the daa-meganizer tool which will further filter the blast results after running DIAMOND. In more detail, blast hits with a bit score below 50, and hits outside the top 10% of the highest bit score were excluded. The meganized .daa files were combined into one .megan report file using the tool compute-comparison (setting: absolute counts) supplied with MEGAN. The data was then imported to MEGAN and analyzed further. On average 20.0 million reads (min 4.4, max 32.5) had been classified and were imported into MEGAN. The lowest read count of 4.4 million was attributed to the sample from station 49 (region Dead Zone Mid-South), however there was no consistent pattern that Dead Zone samples had lower read counts than the rest of the samples (Data S2). On average 4.9 million reads (min 1.0, max 7.9) had been linked to 12,423 unique KEGG KO identifiers affiliated with a known KEGG PATHWAY identifier (see Data S2 and Data S3 for full details and classifications per sample). The KEGG data was extracted and normalized between samples as counts per million values (CPM, i.e., relative proportion × 1,000,000).

### Bioinformatic analyses—metagenome assembled genomes (MAGs)

The quality trimmed reads were used to construct a metagenome co-assembly using MEGAHIT 1.2.9 with default settings [46]. The assembly consisted of 64,413,852 contigs with an average length of 681 bp (min 200, max 403,515). This was followed by “binning” the assembly into metagenome assembled genomes (MAGs) using METABAT 2.12.1 with default settings [47]. This yielded 2216 MAGs which was further analyzed for completeness and contamination using the software CheckM 1.1.3 using the default lineage_wf pipeline with the standard 43 single copy marker genes (SCMG) set [48]. The final data was delimited to MAGs ≥ 95% completeness and ≤ 5% contamination, which resulted in complete or near-complete 46 MAGs. The quality trimmed reads were mapped to the metagenome co-assembly using Bowtie2 2.3.5.1, and the sam files were converted to bam and sorted plus indexed with samtools 1.12 [49]. The % mapped reads per metagenome sample for each MAG was estimated with the CheckM coverage and profile commands. The MAGs were taxonomically and functionally annotated using DIAMOND + MEGAN as described in Bağcı C, Patz S and Huson DH [50]. DIAMOND was run in frame-shift-aware alignment mode (settings: -F 15 --range-culling). The output .daa files were linked to taxonomical (NCBI) and functional (KEGG) annotations using the MEGAN supplied daa-meganizer tool (setting: --longReads). The taxonomy and KEGG data were retrieved from each MAG using MEGAN (see Data S4 for full details and results of % mapped reads and KEGG classifications). In addition, prodigal 2.6.3 [51] was used with default settings to predict genes in each MAG followed by gene classification using BLASTP with a 0.001 *e*-value threshold against the NCBI NR database (database date: September 1, 2021) (Data S4). Metabolic distances between the high-quality MAGs were calculated following the method described by Giri S, Oña L, Waschina S, Shitut S, Yousif G, Kaleta C and Kost C [52], except that here we used Jaccard distances rather than Euclidean. In brief, a metabolic network model containing the 46 high-quality MAGs was constructed using the gapseq 1.1 tool [53] with default settings. Columns with zero metabolic reactions were removed, metabolic distances were power transformed (^2), and Jaccard distances calculated between each MAGs. The metabolic distances between MAGs were compared by grouping MAGs, based on the % mapped reads from each station, with different salinities: North (< 5 ppt), South (> 8 ppt) and Dead Zones. In addition, the metabolic distance between MAGs in the North versus the South was also compared. See Data S4 for full details and results on the grouping of each MAG.

### Bioinformatic analyses—taxonomic annotation

The taxonomic annotation of the data closely followed the procedure previously described in Broman E, Zilius M, Samuiloviene A, Vybernaite-Lubiene I, Politi T, Klawonn I, Voss M, Nascimento FJA and Bonaglia S [28]. In brief, the quality trimmed sequences were taxonomically classified using the Kraken2 + Bracken2 combo. Kraken 2.1.0 [54] was used with a paired-end setup (parameter: --paired) and the reads were classified against the NCBI RefSeq database (downloaded: August 1 2020). Bracken 2.6.0 [55, 56] was then used to estimate relative abundances on genera level using settings: -r 135 -l G -t 10 (i.e., bracken database with a read length of 135 bp, classified to genus level, and using a minimum threshold of 10 counts per genus). The bracken reports were then combined into a biom-format file using the python package kraken-biom 1.0.1 (parameters: ---fmt hdf5 -max D --min G) [57]. Finally, the biom data file was converted to a tab delimited table with the python package biom-format 2.1.7 [58]. The final data consisted on average of 4.4 million classified reads (min 1.1, max 7.9) and was analyzed and normalized as relative abundances (%) in the software Explicet 2.10.5 [59].

### Statistics

Shannon’s *H* alpha diversity index was analyzed in the software Explicet by sub-sampling counts to the lowest sample size for the NCBI RefSeq taxonomy data (1,063,881 counts) and KEGG functional gene data (982,326 counts). The analyses were conducted with a bootstrap × 100 setting and the mean is reported. Bray-Curtis dissimilarity index was used to analyze beta diversity, and analyses were performed based on relative abundances (%) for the NCBI RefSeq taxonomy data, CPM values for the KEGG functional gene data, and the % mapped reads per bin for the MAG data. The Bray-Curtis index was calculated and visualized as non-metric multi-dimensional scaling (NMDS) multivariate plots using the software Past 4.05 [60]. PERMANOVA (9999 permutations) tests were conducted in Past 4.05 to infer significant differences in community composition among the sampled regions. Pairwise PERMANOVA comparisons between regions with Bonferroni corrected *p* values were conducted in the software Past. Statistical tests mentioned below were run as two-sided as by default settings in the mentioned R packages using R 4.1.1 [61]. The *bioenv* function in the R vegan 3.6.1 package [62] was used to infer the main contribution environmental drivers affecting the community composition of the KEGG functional gene data (parameter used: method=“spearman”, index=“bray”, metric=c(“euclidean”). In addition, distance-based redundancy analysis (dbRDA; distance = “bray”, 999 permutations) was conducted to infer which environmental variables was significantly influencing the KEGG functional gene and MAG data, using the *capscale* function in the vegan package. The *simper* function, also part of the R vegan package, was run with 999 permutations and used to infer which KEGG functional genes contributed the most to changes in functional gene composition between the studied regions. Note that SIMPER analyses require the difference between the tested groups (i.e., regions in our study) to be significant to be meaningful. The distance difference in meters between regions were obtained from using the R raster 3.4-10 package using the function *pointDistance* [63]. The input longitude and latitude values were based on averages for each region, and the calculated km distance difference was then used with the SIMPER results of between group dissimilarity. Linear models of the KEGG pathways and abiotic variables were computed using the *lm* function in R. Multicollinearity was accounted for by visualizing correlograms and estimating the variance inflation factor using the *vif* function of the linear models in R (with the criteria of vif < 5). For these reasons, the abiotic variables latitude and longitude were excluded as they were collinear with each other and salinity, TC % and TN % as they were collinear with TC (μmol/g) and TN (μmol/g), and TN which was collinear with TC (when running the linear model for the nitrate/nitrite transporter genes TC was substituted with TN). Pearson correlations between the abiotic variables were conducted using the *rcorr* function in the R package Hmisc 4.3.0 and delimited to significant correlations with an *r* value > 0.7 or < -0.7 [64]. Statistical differences of genes between the studied regions were tested with Kruskal-Wallis tests and Dunn multiple comparisons with Benjamini-Hochberg *P* value adjustments using the *dunn.test* function in the dunn.test 1.3.5 R package [65].

## Results

**Fig. 1:**
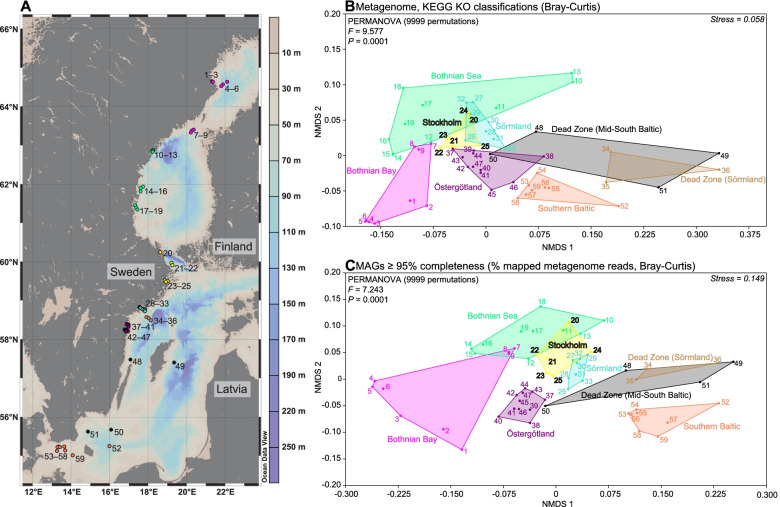
**A** Sediment samples (top 0–2 cm) were collected in Baltic Sea during May–June 2019. The map shows the sampled 59 stations as colored dots according to their specific region (grouped by spatial location; Table 1). The light brown–blue color gradient on the right *y*-axis shows the water depth (m). Dead zone regions are defined by having hypoxic bottom water (< 2 mg/L O_2_) and are colored in brown and black. **B** NMDS showing the beta diversity of the functional genes based on sequenced reads for each station. The figure shows the Bray-Curtis dissimilarity based on KEGG KO identifier read counts normalized as counts per million (CPM) values. The stations are clustered into regions and shown in different colors according to the map and Table 1. **C** NMDS showing the beta diversity (Bray-Curtis) of 46 high quality (≥ 95% genome completeness and ≤ 5% contamination) metagenome assembled genomes (MAGs) for each station. The data is based on the % metagenome mapped reads to the genes in each MAG for each sample. The PERMANOVA results are based on testing all regions together and shows the pseudo-*F* values

Sediment samples (top 0–2 cm layer) were collected during May–June 2019 from 59 stations in the brackish Baltic Sea, that presents conditions close to freshwater (salinity < 2) in the north gradually increasing to ~ 17 in the south (Fig. 1A and Table 1). Stations were sampled for bottom water (water depth, salinity, temperature, O_2_) and sediment chemistry data (TC %, TN %, δ^13^C ‰, δ^15^N ‰) (Table 1 and Data S1). Environmental gradients included, e.g.,: water depth (13–125 m); salinity (2.6–16.2); dissolved O_2_ (0.2–11.5 mg/L); and C/N ratio (7.6–13.8); (see Table 1, Data S1, and Text S1 for full lists of all variables and their statistical analyses). DNA from all sediment samples was extracted, sequenced, and all good-quality reads were analyzed for functional genes, metabolic pathways, and taxonomy. Moreover, assembly and binning recovered 46 high-quality MAGs (≥ 95% completeness) that represented 1.64 % (± 0.14) of the reads.

**Table 1:** The top 0–2 cm sediment layer was collected from distinct sediment cores from 59 stations during May–June 2019 from the north (latitude 64.65300) to south Baltic Sea (latitude 55.00903)

Station	Region	Depth (m)	Salinity	°C	O_2_ mg/L	TN (%)	TC (%)	δ^15^N (‰)	δ^13^C (‰)
1	Bothnian Bay	27	2.6	4.9	10.3	0.1	1.4	3.2	− 25.0
2	Bothnian Bay	37	2.8	3.3	10.4	0.1	1.0	3.1	− 25.7
3	Bothnian Bay	32	2.7	3.9	10.5	0.1	0.7	4.4	− 25.3
4	Bothnian Bay	122	3.7	0.9	9.5	0.4	4.5	4.7	− 25.4
5	Bothnian Bay	106	3.6	1.0	9.9	0.5	5.5	4.7	− 25.8
6	Bothnian Bay	90	3.5	1.3	9.4	0.5	4.9	4.3	− 25.8
7	Bothnian Bay	75	5.8	3.2	7.2	0.3	2.4	4.0	− 24.2
8	Bothnian Bay	89	5.9	3.3	6.9	0.4	3.3	4.3	− 24.7
9	Bothnian Bay	85	5.9	3.3	7.0	0.4	3.0	4.4	− 24.5
10	Bothnian Sea	17	5.3	2.9	2.4	0.8	7.3	2.2	− 22.3
11	Bothnian Sea	46	5.4	2.2	7.6	0.4	3.5	3.5	− 23.7
12	Bothnian Sea	63	5.6	2.6	6.8	0.2	1.9	3.5	− 23.4
13	Bothnian Sea	30	5.3	2.0	7.8	0.6	5.3	2.4	− 21.9
14	Bothnian Sea	79	5.5	2.4	7.2	0.3	2.6	4.3	− 24.3
15	Bothnian Sea	68	5.4	2.1	7.8	0.3	2.8	4.1	− 24.3
16	Bothnian Sea	67	5.3	1.9	9.3	0.3	2.9	4.1	− 23.9
17	Bothnian Sea	49	5.1	2.7	8.5	0.4	3.4	3.8	− 24.1
18	Bothnian Sea	70	5.2	2.1	10.0	0.4	3.5	3.8	− 24.5
19	Bothnian Sea	77	5.2	2.3	10.1	0.4	3.3	4.1	− 24.4
20	Stockholm	23	5.2	5.2	8.9	0.6	5.2	3.6	− 23.9
21	Stockholm	121	6.8	2.8	10.8	0.4	3.4	3.7	− 24.3
22	Stockholm	110	6.8	2.8	8.0	0.5	3.9	3.7	− 24.4
23	Stockholm	24	4.2	9.7	11.1	0.7	5.1	4.7	− 22.6
24	Stockholm	62	5.7	4.7	7.8	0.2	1.6	3.5	− 22.6
25	Stockholm	40	4.9	5.6	10.4	0.4	2.7	4.0	− 22.8
26	Sörmland	22	5.8	8.1	11.3	0.8	6.0	4.5	− 22.8
27	Sörmland	40	6.2	4.6	9.5	0.8	5.6	4.1	− 23.5
28	Sörmland	37	6.1	4.8	10.3	0.8	5.7	3.9	− 23.4
29	Sörmland	38	6.3	4.0	9.3	0.6	4.4	4.4	− 23.4
30	Sörmland	44	6.3	4.2	10.4	0.2	1.5	4.1	− 23.3
31	Sörmland	53	7.5	4.3	8.4	0.6	4.0	4.3	− 23.4
32	Sörmland	47	7.4	4.0	7.4	0.6	4.2	4.1	− 23.3
33	Sörmland	59	6.7	4.2	9.6	0.5	3.5	4.1	− 23.7
34	Dead Zone (Sörmland)	79	10.0	5.8	0.3	0.8	6.4	3.0	− 24.5
35	Dead Zone (Sörmland)	78	10.0	5.8	0.5	0.5	4.5	3.4	− 24.9
36	Dead Zone (Sörmland)	125	9.8	6.3	0.5	1.1	9.4	2.8	− 25.1
37	Östergötland	13	5.8	11.0	10.1	0.9	7.1	4.5	− 22.0
38	Östergötland	13	5.6	11.5	10.8	1.1	7.6	4.7	− 21.9
39	Östergötland	17	5.7	11.6	10.5	1.0	7.4	5.0	− 19.9
40	Östergötland	20	5.7	10.2	8.7	1.1	7.8	4.9	− 22.5
41	Östergötland	20	6.1	9.0	10.6	1.2	8.3	4.6	− 21.9
42	Östergötland	39	5.7	5.9	11.5	1.0	7.1	4.4	− 22.6
43	Östergötland	33	5.6	6.8	11.5	0.9	6.6	4.4	− 22.5
44	Östergötland	25	5.6	8.4	11.1	0.8	6.1	4.0	− 22.5
45	Östergötland	32	5.6	7.9	11.3	0.8	6.0	4.0	− 22.5
46	Östergötland	33	5.7	7.8	11.0	1.2	8.1	3.9	− 23.0
47	Östergötland	29	5.7	8.7	11.2	0.8	5.9	4.0	− 22.1
48	Dead Zone (Mid-South)	79	8.7	5.8	0.3	1.1	8.2	3.5	− 23.5
49	Dead Zone (Mid-South)	111	11.6	7.3	0.3	1.8	16.8	3.0	− 21.2
50	Dead Zone (Mid-South)	71	15.2	8.6	1.1	0.6	4.9	3.3	− 25.1
51	Dead Zone (Mid-South)	80	15.1	8.8	0.2	0.7	5.8	3.1	− 24.8
52	Southern Baltic	91	16.2	8.7	0.5	0.7	6.7	3.3	− 24.1
53	Southern Baltic	41	12.3	5.8	5.3	0.2	1.7	4.6	− 23.6
54	Southern Baltic	43	12.5	6.5	7.6	0.8	6.1	4.0	− 23.9
55	Southern Baltic	41	12.8	6.8	6.4	0.4	2.9	4.2	− 23.5
56	Southern Baltic	41	11.7	5.9	5.2	0.4	3.1	4.0	− 23.7
57	Southern Baltic	45	11.9	7.4	8.6	0.8	6.7	4.0	− 23.0
58	Southern Baltic	39	12.4	9.0	9.6	0.2	1.9	4.1	− 22.5
59	Southern Baltic	48	15.0	8.1	8.5	0.7	6.1	4.3	− 22.7

The table shows the stations listed as clusters from north to south (stations 1 to 59, respectively), water depth (m), bottom water salinity, temperature (°C), and dissolved O_2_ (mg/L). Total nitrogen (%), total carbon (%), and isotope ratios (δ^15^N and δ^13^C ‰) were measured on sampled sediment

### Benthic functional gene composition shows strong spatial differentiation influenced by salinity and oxygen availability

**Fig. 2:**
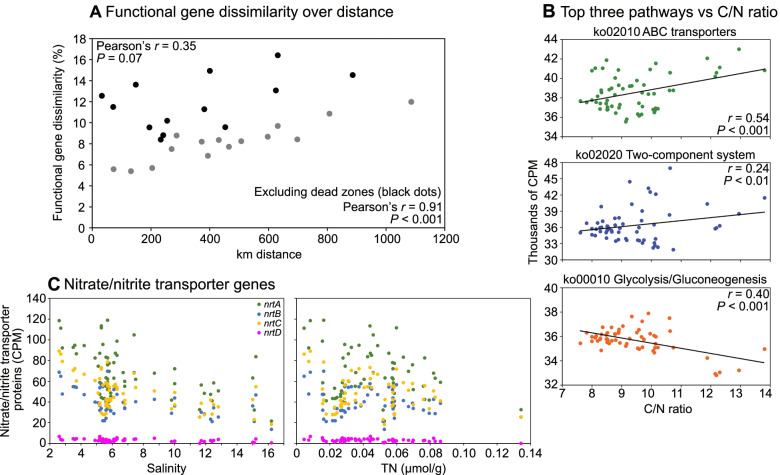
**A** The change in functional genes dissimilarity (%) over km distances in the Baltic Sea. The % dissimilarity between regions were estimated with SIMPER analyses, based on pairwise-test of regions, and distances calculated based in the average latitude and longitude for each region. The data is based on pairwise-tests between regions (i.e., one dot is the distance between two regions) with dead zone regions (Dead Zone (Sörmland) and Dead zone (Mid-South)) denoted as black dots. **B** CPM-values of the top three pathways vs the C/N ratio for all stations. The dashed line shows a linear trendline. **C** CPM-values of the nitrate/nitrite ABC transporter complex *nrtABCD* genes vs salinity and TN

Read-annotation of the whole metagenome dataset yielded on average 8,840 unique KEGG KO gene/protein identifiers per sediment sample (min: 7,759, max: 9,406; see Data S2 for number of reads before quality trimming, after merging of reads, etc.; see Data S3 for all KEGG database hits). There was no correlation in functional gene (KEGG KOs) alpha diversity (Pearson’s *r* = 0.11; 10.89 ± 0.005 Shannon’s *H*; Fig. S1) among our sampled stations, indicating that changes between basins in functional diversity are likely attributed to changes in the composition of genes, rather than the number of different genes. Non-metric multidimensional scaling (NMDS) analysis of the Bray-Curtis dissimilarity of the KEGG KO identifiers showed that the functional gene compositional diversity was different between the regions in the Baltic Sea (PERMANOVA (9999 permutations), pseudo-*F* = 9.6, *P* = 0.0001; Fig. 1B). To further look into detail of some of these differences in functional gene diversity we performed a similar analysis on data from individual MAGs. Interestingly, a difference in beta diversity (Bray-Curtis) was also observed when the representation of functional genes within the high quality MAGs were analyzed between the studied regions (PERMANOVA, pseudo-*F* = 7.24, *P* = 0.0001; Fig. 1C and Data S4). SIMPER analysis revealed a positive correlation between functional gene dissimilarity (%) and the km distance between region with oxic sediments (*r* = 0.91, *P* < 0.001; Fig. 2A; Data S5). To test the difference between regions, stations were considered replicates within their specific region (*n* = 3–12) (see methods for more details). However, this was not evident when including data from the hypoxic (< 2 mg/L O_2_) dead zone regions (*r* = 0.35, *P* > 0.05; Fig. 2A). This shows that that gene dissimilarity was higher when comparing dead zones with oxic regions (up to 16% dissimilarity), while the largest dissimilarity between oxic regions was found when comparing the Bothnian Bay with the Southern Baltic (10% dissimilarity; Data S5). A BIOENV analysis showed that the best explanatory variables for the functional gene compositional diversity (Bray-Curtis) across the 59 stations in the Baltic Sea were latitude, water depth, salinity, oxygen, TC %, C/N, and δ^15^N ‰ (Spearman’s *rho* = 0.69). Similarly, dbRDA analysis showed that salinity, water depth, oxygen, TC %, C/N, temperature, and δ15N ‰ were significantly influencing the functional gene community composition (all *P* < 0.05), as well as the composition of the high-quality MAGs (all *P* < 0.05; full statistics available in Data S6).

### Stoichiometry controls the availability of genes for major metabolic pathways including environmental sensing, membrane transport of nutrients, and carbon metabolism

**Fig. 3:**
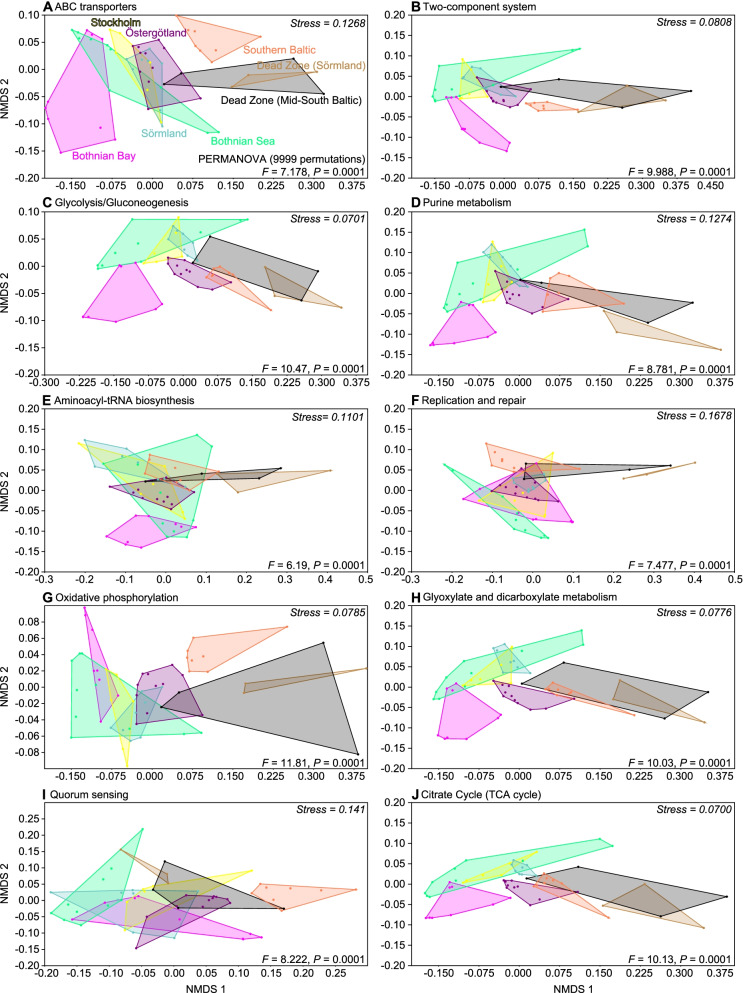
NMDS panels showing the beta diversity (Bray-Curtis dissimilarity) of the functional genes in all reads of the metagenomes within the top 10 KEGG Pathways (based on the average CPM-values for all 59 stations). The metabolic pathways that attributed the highest number of DNA sequences in the Baltic Sea sediments samples are, in order: **A** ABC transporters, **B** two-component system, **C** glycolysis/gluconeogenesis, **D** purine metabolism, **E** aminoacyl-tRNA biosynthesis, **F** replication and repair, **G** oxidative phosphorylation, **H** glyoxylate and dicarboxylate metabolism, **I** quorum sensing, **J** and the citrate cycle (TCA cycle). The PERMANOVA results are based on testing all regions together and shows the pseudo-*F* values

**Fig. 4:**
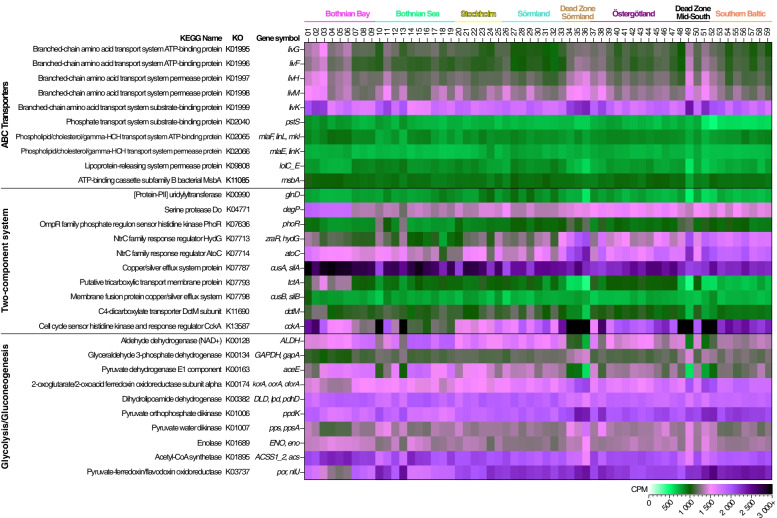
Heatmap showing the top 10 genes within the top three pathways (ABC transporters, two-component system, and glycolysis/gluconeogenesis). The *x*-axis shows the stations with their respective region, and the color gradient is based on CPM-values for each KEGG KO shown on the *y*-axis

The top 10 metabolic pathways in the sediment metagenomes for all stations (based on the average CPM values (counts per million reads) of the KEGG pathways for all stations), were dominated by the ATP-Binding Cassette (ABC) transporters pathway which is crucial in transfer of substrates over the cell membrane (Fig. S2). Other top pathways were associated with environmental sensing and biofilm formation (two-component system and quorum sensing), energy and respiration (e.g., glycolysis/gluconeogenesis and oxidative phosphorylation), and cell maintenance such as replication and repair (Fig. S2). Linear model analyses of the top 10 pathways, with abiotic variables as explanatory variables, showed that variables with the strongest effect (*P* < 0.01) were salinity, water depth, C/N, and δ^15^N (Table S1, see Data S7 full statistical results). Salinity was one of the significant variables for the glycolysis/gluconeogenesis, oxidative phosphorylation, quorum sensing, and replication and repair pathways (*P* < 0.05; Table S1). Looking in more detail at the significant explanatory variables for the top 3 pathways: water depth and C/N were significant for ABC transporters; C/N, TC, and δ^15^N for two-component system; and salinity, C/N, temperature and δ^15^N for glycolysis/gluconeogenesis (Table S1). Taken together, salinity was found to be a significant variable for some specific metabolic pathways used with, e.g., carbohydrate metabolism, but other variables such as the C/N ratio were more important for, e.g., ABC transporters that are used for membrane transport of nutrients (Fig. 2B). Similar to the functional gene dataset (i.e. individual genes not clustered into pathways), the beta diversity of the top 10 dominant pathways also showed a difference between regions (PERMANOVA tests, pseudo-*F* = 6.19–11.81, *P* = 0.0001; Fig. 3). The distinctly different Dead Zone regions likely explained part of the dissimilarity, however beta diversity changed along the spatial gradient even in the oxic sediments for the pathways: ABC transporters, Two-component system, glycolysis/gluconeogenesis, purine metabolism, glyoxylate, and the citrate cycle. In contrast, for the pathways aminoacyl-tRNA biosynthesis and replication and repair clustered more closely among regions (Fig. 3). PERMANOVA pairwise comparisons between the regions for the top 10 pathways listed above showed that the Bothnian Bay and Southern Baltic had the highest number of significant comparisons with other regions (Data S6), indicating that the large difference in salinity at these regions likely influenced the functional gene composition. Salinity was a major variable explaining the gene composition for nutrient uptake as indicated by linear models analyses on the nitrate/nitrite ABC transporter complex *nrtABCD* genes. The results showed there was a lower gene abundance of *nrtA*, *nrtB*, and *nrtC* at higher salinities (*P* < 0.05; Fig. 2C; statistics available in Data S8). A similar pattern was found when *nrtABCD* genes were plotted against TN (μmol/g) but this was not statistically significant in the linear model analysis (Fig. 2C; note the high TN values in the figure from one of the dead zone samples (station 49; Data S1)). Looking closer at the functional gene diversity within the top 3 pathways (ABC transporters, two-component systems, and glycolysis/gluconeogenesis) examples of statistically significant genes include, e.g., similar genes *cusA*/*silA* coding for copper/silver efflux system protein that was higher in the Bothnian Bay and Bothnian Sea when compared to the more southern regions Östergötland, Southern Baltic and both Dead Zones (KW test, df = 7, *H* = 30.4; with Dunn comparison test between regions, Benjamini-Hochberg adjusted *P* values < 0.05; Fig. 4). In contrast, the genes *zrA*/*HydG* coding for a NrtC (nitrogen regulatory protein C) family response regulator was higher in the southern parts of the Baltic (Östergötland and the Southern Baltic compared to both the Bothnian Bay and Sea; KW, df = 7, *H* = 50.0; Dunn test adjusted *P* values < 0.05; Fig. 4). *ALDH* genes coding for Aldehyde dehydrogenases, shown to be used in, e.g., carbon metabolism and osmoprotection [66], had more reads in the mid and northern part of the Baltic, with the Bothnian Bay and Sea, Stockholm and Sörmland all being higher than the Southern Baltic and the Dead Zone regions (KW, df = 7, *H* = 32.9; Dunn test adjusted *P* values < 0.05; Fig. 4). The number of reads attributed to the *pstS* gene coding for a Phosphate transport system substrate-binding protein involved in phosphate import was higher in the Bothnian Bay and Sea, Stockholm, and Östergötland when compared to the Southern Baltic (KW, df = 7, *H* = 32.6; Dunn test adjusted *P* values < 0.05; Fig. 4).

In addition to the major metabolic pathways and their gene diversity, we also investigated the top functional genes in the studied system. Dominant genes in the Baltic Sea sediments included, e.g., a gene coding a large repetitive protein used in biofilm formation, a putative transposase which, are proteins known to be used in genome plasticity, arylsulfatase that is involved in the hydrolysis of sulfur ester-bonds common in algal sulfated polysaccharides, and genes used in, e.g., fatty acid biosynthesis, DNA replication and repair, and metabolism of purines and pyrimidines which are key components in nucleic acids (Fig. S2). Looking closer at a few examples of what genes in the sediment contributed significantly (SIMPER analysis, *P* < 0.05) to the functional gene dissimilarity between the studied regions: the gene *bapA* coding for a large repetitive protein used in biofilm formation had the most attributed reads in the Southern Baltic (Data S5); an unnamed Arylsulfatase gene had more read counts in the mid-Baltic and Southern Baltic, except for the Dead Zone regions (Data S5); the gene *hdrA2* coding for the enzyme “heterodisulfide reductase subunit A” used in methanogenesis was more abundant in the Dead Zone regions (Data S5); and a multidrug efflux pump gene coding for resistance against beta-Lactam antibiotics was higher in the northern Baltic and mid-Baltic (Stockholm and Sörmland) when compared to the Southern Baltic (Data S5).

### Microbial MAGs in dead zones are metabolically more similar than those in oxic sediments

Furthermore, Jaccard distances calculated from metabolic distances of the 46 high-quality MAGs with majority of their mapped reads (%) at the low saline North (< 5), high saline South (> 8), or low oxygen Dead Zones showed that the dead zones had a lower metabolic distance between the MAGs (Kruskal-Wallis (KW) test, df = 3, *H* = 40.9, with multiple comparison Dunn test, *P* < 0.05; Fig. S5 and Data S4). This indicates that genomes are metabolically more similar in dead zones compared to both low- and high-saline regions with oxic sediments in the Baltic. The metabolic distance between MAGs in the North and South also showed a high dissimilarity (Jaccard distance > 0.9), however this was not different from the metabolic distances within the North region (> 0.9; Fig. S5).

### Microbial community composition is also structured by strong changes in salinity and oxygen

**Fig. 5:**
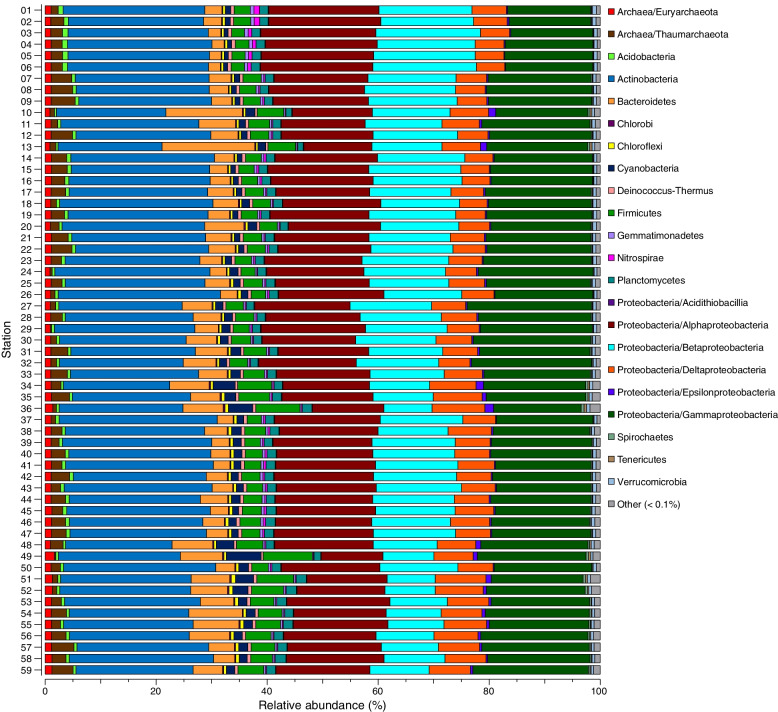
Stacked bars showing the NCBI RefSeq classified taxonomy based on the metagenome dataset. The *y*-axis shows each station as shown in the map in Fig. 1 while the *x*-axis shows the relative abundance (%). The data has been grouped as Phyla or Proteobacteria classes. “Other” contains groups with less than 0.1% average of all samples

Taxonomic classification of the metagenome data on the lowest classified level, i.e., genus (NCBI RefSeq database, Data S9) showed that the Shannon’s *H* alpha diversity index only had a weak correlation with spatial location (*r* = 0.57). Also, there was no large differences between the north and south Baltic (Bothnian Bay 8.40 ± 0.02, Southern Baltic 8.67 ± 0.02 Shannon’s *H*) (Fig. S3). The Bray-Curtis beta diversity of the microbial community showed a statistical significance (PERMANOVA, pseudo-*F* = 8.47, *P* = 0.0001); however, the pattern along our regional spatial gradient was not as clear as the functional gene data. Instead, the largest differences in the Bray-Curtis diversity were related to strong changes in salinity and oxygen: the north (Bothnian Bay), Southern Baltic, and Dead Zone areas (Fig. S4). The dataset showed that the relative abundance of bacterial phyla was similar across our Baltic Sea stations (including dead zone areas), with only minor differences (Fig. 5). Actinobacteria and Proteobacteria had the highest relative abundance (%) that together represented 80.28 ± 1.20% of the dataset (based on all 59 stations; Fig. 5). These two groups showed minor variation along the Baltic Sea, with Actinobacteria (24.22 ± 0.003%), Proteobacteria classes gamma (18.27 ± 0.002%), alpha (17.27 ± 0.002%), beta (13.93 ± 0.003%), and Delta (6.45 ± 0.001%; Fig. 5). On genus level, three genera among the most abundant taxa were distinctly different either increasing, decreasing, or showing a high variation in their relative abundance, across the Baltic Sea spatial gradient. These were *Nitrosopumilus*, *Burkholderia*, and *Desulfosarcina* (Fig. S6). Our abiotic variables were unable to explain the variation in the archaeal nitrifier *Nitrosopumilus* (Spearman correlations, *P* > 0.05), however salinity correlated negatively with Betaproteobacteria *Burkholderia* (*rho* = − 0.65, *P* < 0.0001) which had the highest relative abundance in the north Baltic. Conversely, salinity correlated negatively with the sulfate-reducing bacteria *Desulfosarcina* (*rho* = 0.76, *P* < 0.0001) which had the highest relative abundance in the south Baltic Sea (Fig. S6). Finally, BIOENV analysis on genus level showed that the best combination of explanatory variables for the community composition (Bray-Curtis) included latitude, salinity, oxygen, TC %, C/N, and δ15N ‰ (Spearman’s rho = 0.72).

## Discussion

We have shown here that the composition of functional genes, metabolic pathways, and microbial communities in the seafloor spanning over 1000 km distance is structured not only by environmental conditions like salinity and oxygen, but also by carbon and nitrogen origin and availability. Functional gene dissimilarity increased by geographic distance, and salinity was one of the main drivers of functional gene composition in sediment. Sediments located in higher saline water are known to harbor a higher biodiversity of benthic micro- and macrofauna [15, 16]; however, this pattern was not observed for pelagic prokaryotes in the Baltic Sea [14]. While a higher species richness of prokaryotic operational taxonomic units (OTUs) based on rRNA has been detected in the mid-Baltic compared to the south and north [67], we did not find a difference in alpha diversity for both microbial genera or functional genes in the sediments. These findings are in contrast to our hypothesis that lower oxygen and salinity would decrease diversity and limit functional capabilities. Our findings are in accordance with studies that found salinity to influence the gene composition of sediment microbial communities in systems such as lakes and estuaries [68, 69]. For example, a study conducted in the Hangzhou Bay estuary identified salinity to be one of the most influential variables for gene categories related to, e.g., phosphorous, sulfur, and nitrogen cycling [68], while another study found that salinity was an important factor shaping microbial communities in the Baltic Sea [70], and Qinghai-Tibetan Plateau lake sediments [69]. In our study, major pathways such as ABC transporters, two-component systems, and glycolysis/gluconeogenesis had a gene composition that changed along the spatial gradient, seemingly controlled by variables related to resource availability (e.g., C/N, TC, and δ^15^N) in addition to salinity and temperature as indicated by the linear models. However, nutrient uptake transporters such as the nitrate/nitrite transporter genes *nrtABCD* were significantly explained by salinity but not TN, indicating that salinity had a larger influence on these ABC transporter genes than the availability of nitrogen in the sediment. These spatial differences in gene abundance of major pathways suggest potential consequences for carbon cycling. Indeed, high variability of organic carbon recycling rates within the Baltic Sea basins has been previously reported, with the mid-Baltic (i.e., Baltic Proper) showing higher recycling rates compared to the northern Baltic [71]. Moreover, the Baltic Proper has a history of stronger exposure to eutrophication compared to the northern basins [72], and together these differences in carbon recycling and nutrient load might partly explain changes in gene composition related to carbon cycling and nutrient membrane transport in our dataset. Additionally, salinity has been shown to be a factor promoting sulfate reduction and biofilm formation in sediments [73], and partly explaining why the relative abundance of sulfate reducing *Desulfosarcina* and genes for biofilm function were more prevalent in the saline southern Baltic. As suggested by the clustering of stations within the studied regions in the NMDS plots, our results indicate that environmental variables like oxygen, temperature, organic matter, and nutrients have a larger influence on community and gene composition than relatively stable parameters such as salinity [10, 74, 75]. We found essential pathways such as Aminoacyl-tRNA biosynthesis and replication and repair to cluster more closely in the NMDS plots among regions (compared to the pathways described above). Synthesis of Aminoacyl-tRNA is essential in protein synthesis for living organisms as Aminoacyl-tRNAs are involved in the ligation of amino acids to tRNAs [76]. Replication and repair contain coding genes such as DNA helicases and polymerases used in, e.g., transcription and DNA repair plus replication [77]. Finally, because the higher eutrophication in the Baltic Proper [72], the large deposition of algal matter on the seafloor could explain why the Baltic Proper and southern Baltic had more genes for potential degradation of algal sulfated lipids (i.e., arylsulfatase) [78] compared to the northern Baltic. Taken together our results indicate that sediment stoichiometry (such as the C:N ratio), salinity, and oxygen concentration influence functional gene composition and control the availability of genes for major metabolic pathways in sediments.

Because of the aforementioned extensive eutrophication with subsequent decreased oxygen availability in the bottom waters [79], the Baltic Sea has today one of the largest dead zones in the world [80]. Metabolic pathways were clearly impacted in our dead zones sediments as also indicated by the role of oxygen in driving functional gene composition. In an environment that favors anaerobic metabolic processes, with a large range of associated electron acceptors, dead zones are metabolically different from oxic sediments [1]. This was also reflected in the metabolic distance analyses of the high-quality MAGs, which suggested that dead zones contain genomes that are metabolically more similar between each other than when compared to genomes in oxic sediments. Oxygen manipulations of sediments changed the prokaryotic alpha diversity (both increase and decrease) [10, 81], however in our dataset we did not observe a large difference in alpha diversity between oxic station and stations with long-term anoxic sediments. A possible reason is our use of metagenome sequencing compared to the more common amplicon sequencing approach used in those studies to estimate alpha diversity. Dead zone sediments were some of the most dissimilar sediments in terms of functional gene composition across the Baltic Sea. Since many Baltic Sea dead zones have been in that state for more than 50 years, and have stable environmental conditions below a permanent halocline [79] it is possible that these microbial communities are an effect of selective survival [82] or adaptive evolution. The long-term anoxic conditions selecting for anaerobic processes in these sediments likely explain why these zones had the most dissimilar functional gene composition and most metabolically similar MAGs when compared to oxic areas. Finally, we found that δ^15^N (‰) explained part of the gene composition in the dataset, with δ^15^N (‰) values closer to 0 being an indicator of higher organic matter content derived from N_2_-fixation such as by cyanobacteria [83]. These findings explain why the Dead Zones stations had lower δ^15^N (‰) values as these areas can accumulate large quantities of algal material, including diazotrophic organisms [9, 79, 84] that potentially remains longer in the sediment because degradation is slower under anoxic conditions [85, 86]. These findings indicate that the expansion of oxygen deficient waters have long-term effects on benthic microbial communities and the composition of functional genes.

Climate change is currently altering biological, chemical, and physical factors in the oceans and coastal ecosystems [87]. The relevant studied environmental parameters here, such as changes in salinity, decrease in oxygen, and eutrophication are major threats to biodiversity [88]. Biodiversity supports ecosystems services such as food availability and provision of clean water, but also ecosystems processes including, e.g., nutrient cycling [88]. However, even though the alpha diversity of benthic meiofauna (animals < 1 mm) and macrofauna are pronounced over environmental gradients [15, 16], this was not obvious for the microorganisms or their functional gene composition. It can be expected that in sediments, which include a redox cascade with both aerobic and anaerobic metabolic pathways [1], are especially rich in the repertoire of functional genes. The large variety of metabolic capabilities might therefore persist in benthic habitats under environmental change (i.e., core functions are preserved) [22]; however, anthropogenically stressed areas might have less/more abundance of some functional genes limiting the potential of related metabolic processes. Interestingly, according to our multivariate analyses this compositional change in functional genes is more prominent across the studied gradients than taxonomic composition. The composition of bacterial phyla in our dataset was essentially constant across the salinity gradient in the Baltic, but based on previous findings using amplicon sequencing larger differences in phyla are expected at higher salinities close to a fully marine setting [67]. Considering that sinking particles from the water column harbors microbial communities [29, 30] and that the Baltic Sea is a relatively shallow system (average water depth 56 m) [89] it is likely that a portion of microbes in the sediment have been recruited from the water column. This possible recruitment from the water column can to some extent influence the functional gene diversity in the sediment surface. However, it remains to be studied to what extent such sinking particles contribute to functional gene diversity and abundance. Moreover, because of the large number of functional genes in sediments compared to microbial genera, changes in functional gene abundances in response to environmental gradients might be easier to detect. However, it is possible that marine bacteria can adapt to different environmental conditions via selective loss of genes (genomic streamlining) or acquire traits from other bacteria [90, 91]. Our data suggests that that functional gene diversity, rather than taxonomic diversity, is a determining driver of microbial adaptation to local environmental conditions in benthic habitats. Amplicon-based PCR approaches used to investigate microbial taxonomy, although very useful for the study of biodiversity and community structure responses, might not fully elucidate functional patterns solely by comparing taxonomy with geochemical data. Future impacts of climate change with alterations in biogeochemical cycles and effects, like water deoxygenation, increased freshwater runoff, and enhanced eutrophication [5–8], are therefore expected to alter functional gene composition and metabolic pathways in benthic habitats of the inhabiting sediment microbial communities.

## Conclusions

We found that the composition of functional genes was driven by gradients in salinity, oxygen, and carbon and nitrogen at the regional scale of the Baltic Sea. This change in functional genes over the environmental gradients was more prominent than changes in microbial genera, and indicate that functional diversity is an important mediator of adaption to different local environmental conditions. Compared to oxic sediments, oxygen deficient areas had a higher gene dissimilarity and metabolically more similar MAGs. Our findings indicate that natural or anthropogenic changes in, e.g., oxygenation, salinity, and carbon plus nitrogen content will alter functional gene composition and metabolic pathways in benthic habitats.

## Supplementary Information

**Additional file 1: Data S1**. Station numbers with corresponding region, stations, sampling date, WGS84 coordinates, and measured values for the abiotic variables.

**Additional file 2: Data S2**. Bioinformatic information such as Sequencing facility sample IDs, station number, region, and station label according to the yearly monitoring programme, number of sequences yielded before and after quality trimming, average read lengths, Phred33 quality scores, sequences classified with kraken2 + bracken2 against NCBI RefSeq, and the number of KEGG classified reads with DIAMOND + MEGAN.

**Additional file 3: Data S3**. KEGG classifications with station numbers (1 to 59) corresponding to each sample as listed in Table 1. The first sheet shows normalized read counts as CPM values, while the second sheet shows absolute counts.

**Additional file 4: Data S4**. Sheet 1 shows the % metagenome reads mapped to each MAG for each sample (i.e. station in the y-axis). Red cells denote the samples where the MAG was detected. Sheet 2 shows the KEGG classifications (using MEGAN) for each MAG. Sheet 3 shows the NCBI NR classifications (using BLASTP) for each MAG. Sheet 4 shows the % metagenome reads mapped to the MAGs averaged per region. Sheet 5 shows the % average metagenome reads mapped for each MAG based on the salinity and dead zones. The grouping of each MAG into a salinity group was based on the salinity range where they had the highest % mapped reads. Sheet 6 shows the Jaccard distances calculated from the metabolic distances between MAGs in each group shown in Sheet 5.

**Additional file 5: Data S5**. SIMPER analysis results of all KEGG KO identifiers for all regions tested against each other. Sheet 1 shows the top 10 output results for each pairwise test, with red text denoting KEGG KO identifiers with a significant dissimilarity between the tested regions (*P* < 0.05). Sheet 2 shows the group dissimilarity (%) for each region compared to their distance (km) between each other.

**Additional file 6: Data S6**. Full statistical results from the dbRDA analyses in R of the KEGG functional gene data and high-quality MAGs.

**Additional file 7: Data S7**. Pairwise PERMANOVA (9999 permutation) test between regions based on the Bray-Curtis dissimilarity index. The data was tested for the top 10 pathways. The p-values shown are Bonferroni corrected for multiple comparisons.

**Additional file 8: Data S8**. Full statistical results from linear model analyses in R of the top 10 KEGG pathways plus abiotic variables (collinear variables removed, see methods for more details). The second shows the results from the linear model analysis of the *nrtABCD* genes.

**Additional file 9: Data S9**. NCBI RefSeq prokaryotic taxonomic classifications with station numbers (1 to 59) corresponding to each station as listed in Table 1. The first sheet shows normalized read counts as relative abundance (%), while the second sheet shows absolute counts.

**Additional file 10: Figure S1**. Shannon’s H of all KEGG KOs for each station. The data was normalized by sub-sampling to the lowest read count (982,326). Each dot in the graphs shows the mean after bootstrap × 100. **Figure S2**. A) Top ten metabolic pathways, and B) functional genes in the Baltic Sea sediment samples (KEGG KO hits with their respective KEGG pathway shown in parenthesis). KEGG KO identifiers classified to the “Function unknown” pathway or unclassified metabolism in the “Enzymes with EC numbers” pathway was not included in subpanel (A). The data shown is based on the average CPM-values for all 59 stations (±SE). **Figure S3**. Shannon’s H of the NCBI RefSeq taxonomy based for each station. The data was normalized by sub-sampling to the lowest read count (1,063,881). Each dot in the graphs shows the mean after bootstrap × 100. **Figure S4**. NMDS showing the beta diversity (Bray-Curtis dissimilarity) of the microbial community (NCBI RefSeq classified taxonomy) at the lowest classified level, i.e. genus. The data was normalized as relative abundances (%). The PERMANOVA results are based on testing all regions together and shows the pseudo-*F* value. **Figure S5**. The figure shows the Jaccard distance (y-axis) based on the metabolic distance between the high-quality MAGs in different areas of the Baltic Sea. The MAGs were grouped according to salinity as North (<5), South (>8), or Dead Zones. The metabolic distance between MAGs present in the North and South were also calculated and is shown as “North vs South”. The error bars show SE. **Figure S6**. Heatmap showing the top relative abundant (%, color legend shown on the y-axis) classified hits on the lowest taxonomic level (genus) based on the NCBI RefSeq data. The heatmap is delimited to only show genera > 0.5% average of all samples. The x-axis shows the results for each station. **Table S1**. Results from the linear models of the top 10 pathways. The CPM is based on the average CPM values for all station (*n* = 59) as shown in Fig. S1. The stars denote: * = *P* < 0.05; ** = *P* < 0.01 ; *** = *P* < 0.001. **Text S1**. Correlations between abiotic variables.

## Data Availability

All data analyzed in this study are available in the Supplementary Data and the raw sequence data is available online at the European Nucleotide Archive accession number PRJEB41834. https://www.ncbi.nlm.nih.gov/bioproject/?term=PRJEB41834
