# Supplementary material for: Microbial functional genes are driven by gradients in sediment stoichiometry, oxygen, and salinity across the Baltic benthic ecosystem
Source: Microbiome. 2022 Aug 15;10:126. doi: 10.1186/s40168-022-01321-z (PMC9377124; doi:10.1186/s40168-022-01321-z)
Supplement: Supplementary file 11 — Additional file 10: Figure S1. Shannon’s H of all KEGG KOs for each station. The data was normalized by sub-sampling to the lowest read count (982,326). Each dot in the graphs shows the mean after bootstrap × 100. Figure S2. A) Top ten metabolic pathways, and B) functional genes in the Baltic Sea sediment samples (KEGG KO hits with their respective KEGG pathway shown in parenthesis). KEGG KO identifiers classified to the “Function unknown” pathway or unclassified metabolism in the “Enzymes with EC numbers” pathway was not included in subpanel (A). The data shown is based on the average CPM-values for all 59 stations (±SE). Figure S3. Shannon’s H of the NCBI RefSeq taxonomy based for each station. The data was normalized by sub-sampling to the lowest read count (1,063,881). Each dot in the graphs shows the mean after bootstrap × 100. Figure S4. NMDS showing the beta diversity (Bray-Curtis dissimilarity) of the microbial community (NCBI RefSeq classified taxonomy) at the lowest classified level, i.e. genus. The data was normalized as relative abundances (%). The PERMANOVA results are based on testing all regions together and shows the pseudo-F value. Figure S5. The figure shows the Jaccard distance (y-axis) based on the metabolic distance between the high-quality MAGs in different areas of the Baltic Sea. The MAGs were grouped according to salinity as North (<5), South (>8), or Dead Zones. The metabolic distance between MAGs present in the North and South were also calculated and is shown as “North vs South”. The error bars show SE. Figure S6. Heatmap showing the top relative abundant (%, color legend shown on the y-axis) classified hits on the lowest taxonomic level (genus) based on the NCBI RefSeq data. The heatmap is delimited to only show genera > 0.5% average of all samples. The x-axis shows the results for each station. Table S1. Results from the linear models of the top 10 pathways. The CPM is based on the average CPM values for all station (n = 59) as shown in [file 40168_2022_1321_MOESM11_ESM.docx]

**Microbial functional genes are driven by gradients in sediment stoichiometry, oxygen, and salinity across the Baltic benthic ecosystem**

Elias Broman, Dandan Izabel-Shen, Alejandro Rodríguez-Gijón, Stefano Bonaglia, Sarahi L. Garcia, Francisco J.A. Nascimento

**Supplementary Information**

*Data file captions*

**Data S1** Station numbers with corresponding region, stations, sampling date, WGS84 coordinates, and measured values for the abiotic variables. xlsx

**Data S2** Bioinformatic information such as Sequencing facility sample IDs, station number, region, and station label according to the yearly monitoring programme, number of sequences yielded before and after quality trimming, average read lengths, Phred33 quality scores, sequences classified with kraken2+bracken2 against NCBI RefSeq, and the number of KEGG classified reads with DIAMOND+MEGAN. xlsx

**Data S3** KEGG classifications with station numbers (1 to 59) corresponding to each sample as listed in Table 1. The first sheet shows normalized read counts as CPM values, while the second sheet shows absolute counts. Xlsx

**Data S4** Sheet 1 shows the % metagenome reads mapped to each MAG for each sample (i.e. station in the y-axis). Red cells denote the samples where the MAG was detected. Sheet 2 shows the KEGG classifications (using MEGAN) for each MAG. Sheet 3 shows the NCBI NR classifications (using BLASTP) for each MAG. Sheet 4 shows the % metagenome reads mapped to the MAGs averaged per region. Sheet 5 shows the % average metagenome reads mapped for each MAG based on the salinity and dead zones. The grouping of each MAG into a salinity group was based on the salinity range where they had the highest % mapped reads. Sheet 6 shows the Jaccard distances calculated from the metabolic distances between MAGs in each group shown in Sheet 5.

**Data S5** SIMPER analysis results of all KEGG KO identifiers for all regions tested against each other. Sheet 1 shows the top 10 output results for each pairwise test, with red text denoting KEGG KO identifiers with a significant dissimilarity between the tested regions (*P* < 0.05). Sheet 2 shows the group dissimilarity (%) for each region compared to their distance (km) between each other. Xlsx

**Data S6** Full statistical results from the dbRDA analyses in R of the KEGG functional gene data and high-quality MAGs.

**Data S7** Pairwise PERMANOVA (9999 permutation) test between regions based on the Bray-Curtis dissimilarity index. The data was tested for the top 10 pathways. The p-values shown are Bonferroni corrected for multiple comparisons.

**Data S8** Full statistical results from linear model analyses in R of the top 10 KEGG pathways plus abiotic variables (collinear variables removed, see methods for more details). The second shows the results from the linear model analysis of the *nrtABCD* genes. xlsx

**Data S9** NCBI RefSeq prokaryotic taxonomic classifications with station numbers (1 to 59) corresponding to each station as listed in Table 1. The first sheet shows normalized read counts as relative abundance (%), while the second sheet shows absolute counts. xlsx

*Supplementary figures*


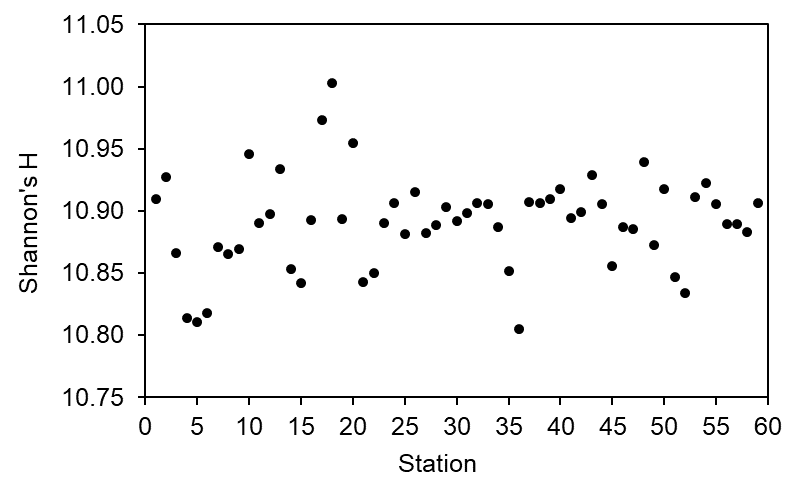


**Figure S1** Shannon’s H of all KEGG KOs for each station. The data was normalized by sub-sampling to the lowest read count (982,326). Each dot in the graphs shows the mean after bootstrap × 100.


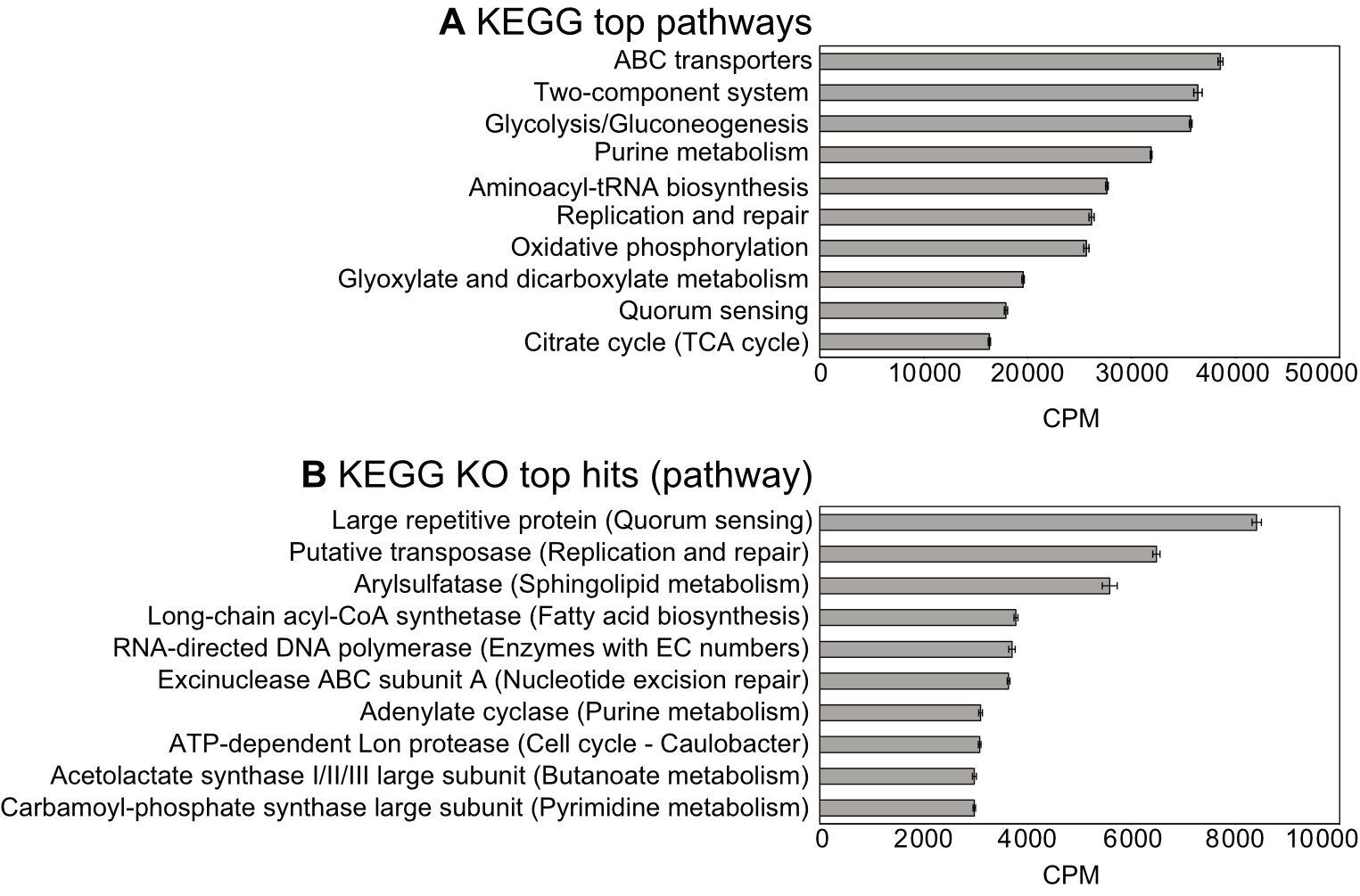


**Figure S2** A) Top ten metabolic pathways, and B) functional genes in the Baltic Sea sediment samples (KEGG KO hits with their respective KEGG pathway shown in parenthesis). KEGG KO identifiers classified to the “Function unknown” pathway or unclassified metabolism in the “Enzymes with EC numbers” pathway was not included in subpanel (A). The data shown is based on the average CPM-values for all 59 stations (± SE).

**
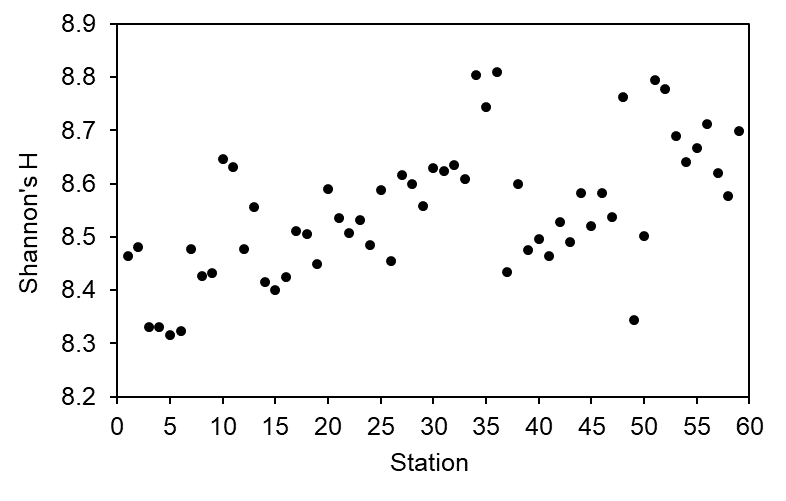
**

**Figure S3** Shannon’s H of the NCBI RefSeq taxonomy based for each station. The data was normalized by sub-sampling to the lowest read count (1,063,881). Each dot in the graphs shows the mean after bootstrap × 100.

**
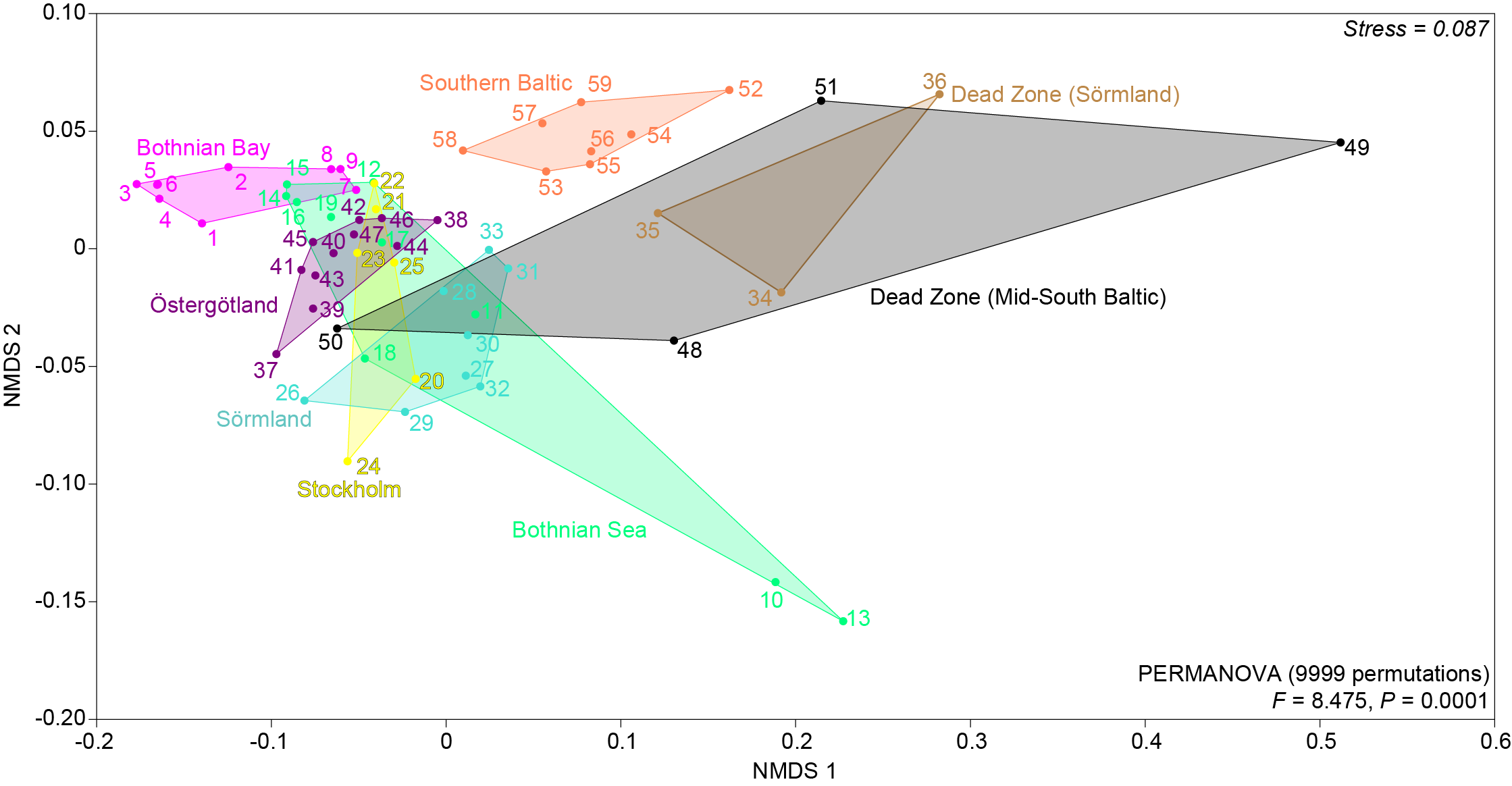
**

**Figure S4** NMDS showing the beta diversity (Bray-Curtis dissimilarity) of the microbial community (NCBI RefSeq classified taxonomy) at the lowest classified level, i.e. genus. The data was normalized as relative abundances (%). The PERMANOVA results are based on testing all regions together and shows the pseudo-*F* value.


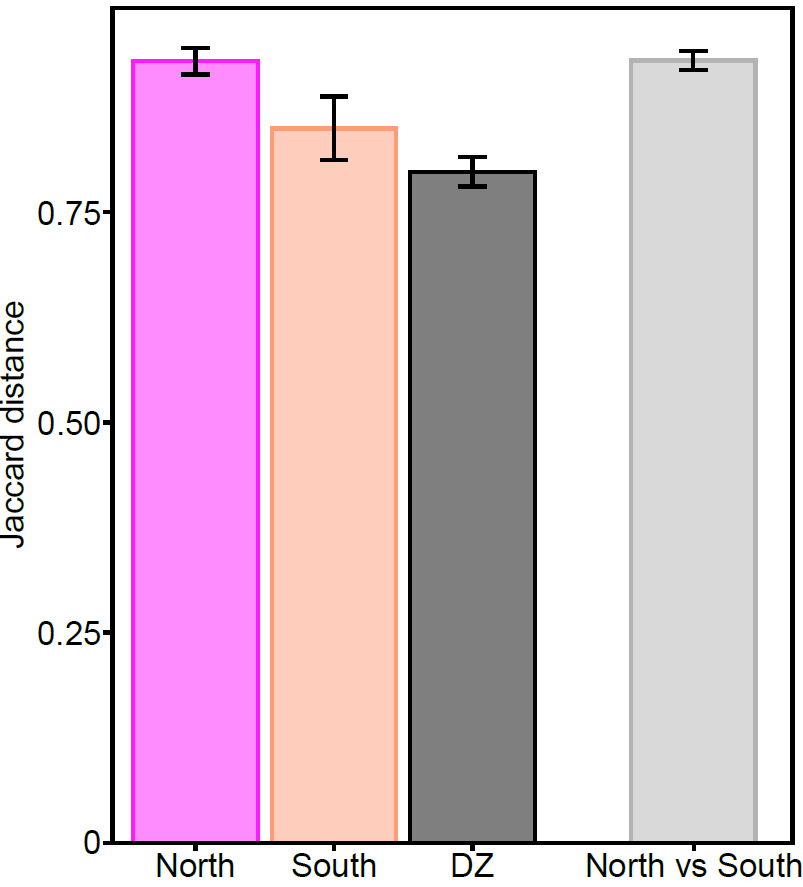


**Figure S5** The figure shows the Jaccard distance (y-axis) based on the metabolic distance between the high-quality MAGs in different areas of the Baltic Sea. The MAGs were grouped according to salinity as North (<5), South (>8), or Dead Zones. The metabolic distance between MAGs present in the North and South were also calculated and is shown as “North vs South”. The error bars show SE.


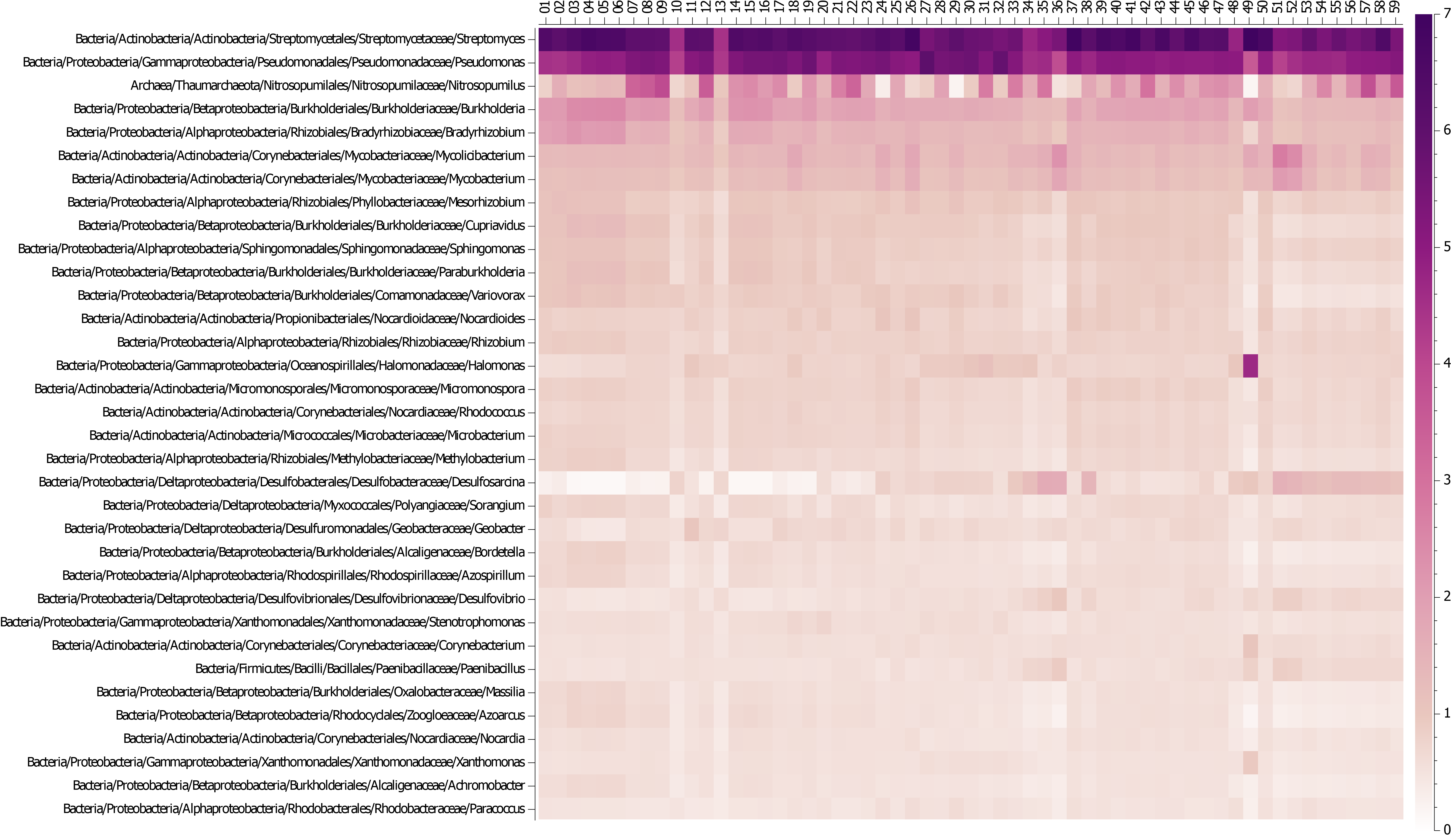


**Figure S6** Heatmap showing the top relative abundant (%, color legend shown on the y-axis) classified hits on the lowest taxonomic level (genus) based on the NCBI RefSeq data. The heatmap is delimited to only show genera > 0.5% average of all samples. The x-axis shows the results for each station.

*Supplementary tables*

|  | ABC trans | Two-comp | Glycolysis | Purine met | Aminoacyl-tRNA | Rep and repair | Oxidative phos | Glyoxylate met | Quorum sensing | Citrate cycle |
| --- | --- | --- | --- | --- | --- | --- | --- | --- | --- | --- |
| Salinity |  |  | ** |  |  | * | ** |  | *** |  |
| Water depth | * |  |  |  | ** | *** |  |  |  |  |
| C/N | *** | ** | *** |  |  |  |  |  | * |  |
| TC |  | * |  |  |  |  | * | ** |  | ** |
| Temperature |  |  | ** |  |  | ** |  |  |  | * |
| Oxygen |  |  |  |  |  |  |  |  |  | * |
| δ15N |  | *** | *** | * |  |  |  | *** |  | *** |
| δ13C |  |  |  |  |  |  |  |  |  |  |

**Table S1** Results from the linear models of the top 10 pathways. The CPM is based on the average CPM values for all station (*n* = 59) as shown in Figure S1. The stars denote: * = *P* < 0.05; ** = *P* < 0.01 ; *** = *P* < 0.001.

*Text S1: Correlations between abiotic variables*

Based on bottom water chemistry data from the 59 stations sampled and correlations (limited to: Pearson’s *r*-values > 0.7 or < -0.7), salinity was negatively correlated with latitude with the lowest salinity in the north and highest salinity in the southern Baltic Sea (from 2.6 to 16.2 ppt); *r* = -0.79, *P* < 0.001). Similarly, temperature was negatively correlated with latitude with the warmest bottom water in the Baltic Proper and southern Baltic Sea (from 0.9 in the north to 11.6°C in mid-Baltic Proper; *r* = -0.70, *P* < 0.001). To see if this was related to the difference in time of sampling, temperature and sampling date (Julian date) was tested with a correlation which indicated that there was no relationship (*r* = 0.04). That organic matter load in the sediment increased the TC and TN content was confirmed with sediment TC % correlating positively with TN % (*r* = 0.97, *P* < 0.001), and that both TC % and TN % correlated positively with TC and TN (*r* = 0.75–0.99, *P* < 0.001). However, sediment δ^13^C and δ^15^N did not correlate strongly with any variables, with the most significant correlations including δ^13^C with temperature and δ^15^N with O_2_ (both *r* = 0.60, *P* < 0.001). Almost all stations were normoxic except stations within known Dead Zone regions (defined as < 2 mg/L O_2_). Water depth was found to have a weak correlation with O_2_ (*r* = -0.46, *P* < 0.001) likely explained by the well aerated bottom waters in the northern Baltic, considering that the halocline in the more saline mid-south Baltic is associated with low oxygen areas and dead zone formation.
